# Formation of body appendages during caudal regeneration in *Platynereis dumerilii*: adaptation of conserved molecular toolsets

**DOI:** 10.1186/s13227-016-0046-6

**Published:** 2016-04-12

**Authors:** Jan Grimmel, Adriaan W. C. Dorresteijn, Andreas C. Fröbius

**Affiliations:** Institut für Allgemeine und Spezielle Zoologie, Abteilung Entwicklungsbiologie, Justus-Liebig-Universität Gießen, Stephanstraße 24, 35390 Gießen, Germany

**Keywords:** *Platynereis*, Regeneration, Body appendages, *Homothorax*, *Extradenticle*, *Distal*-*less*, *Decapentaplegic*, *Buttonhead*

## Abstract

**Background:**

*Platynereis* and other polychaete annelids with homonomous segmentation are regarded to closely resemble ancestral forms of bilateria. The head region comprises the prostomium, the peristomium, a variable number of cephalized body segments and several appendages, like cirri, antennae and palps. The trunk of such polychaetes shows numerous, nearly identical segments. Each segment bears a parapodium with species-specific morphology on either side. The posterior end of the trunk features a segment proliferation zone and a terminal pygidium with the anus and anal cirri. The removal of a substantial part of the posterior trunk is by no means lethal. Cells at the site of injury dedifferentiate and proliferate forming a blastema to regenerate both the pygidium and the proliferation zone. The pygidium forms new anal cirri, and the proliferation zone generates new segments at a rapid pace. The formation of body appendages like the cirri and the segmental parapodia can thus be studied in the caudal regenerate of *Platynereis* within only a few days.

**Results:**

The development of body appendages in *Platynereis* is regulated by a network of genes common to polychaetes but also shared by distant taxa. We isolated DNA sequences from *P. dumerilii* of five genes known to be involved in appendage formation within other groups: *Meis/homothorax*, *Pbx1/extradenticle*, *Dlx/Distal*-*less*, *decapentaplegic* and *specificprotein1*/*buttonhead.* Analyses of expression patterns during caudal regeneration by in situ hybridization reveal striking similarities related to expression in arthropods and vertebrates. All genes exhibit transient expression during differentiation and growth of segments. As was shown previously in other phyla *Pdu*-*Meis/hth* and *Pdu*-*Pbx1/exd* are co-expressed, although the expression is not limited to the proximal part of the parapodia. *Pdu*-*Dll* is prominent in parapodia but upregulated in the anal cirri. No direct dependence concerning *Pdu*-*Dll* and *Pdu*-*sp/btd* expression is observed in *Platynereis*. *Pdu*-*dpp* shows an expression pattern not comparable to its expression in other taxa.

**Conclusions:**

The expression patterns observed suggest conserved roles of these genes during appendage formation across different clades, but the underlying mechanisms utilizing this toolset might not be identical. Some genes show broad expression along the proximodistal axis indicating a possible role in proximodistal patterning of body appendages. Other genes exhibit expression patterns limited to specific parts and tissues of the growing parapodia, thus presumably being involved in formation of taxon-specific morphological differences.

**Electronic supplementary material:**

The online version of this article (doi:10.1186/s13227-016-0046-6) contains supplementary material, which is available to authorized users.

## Background

Across the animal kingdom, body appendages have evolved in various phyla. Morphology of individual appendages has been adapted according to their function. During morphogenesis, the position at which a new appendage is supposed to form is determined and additional body axes, the proximodistal axes, of the new appendages are established. Concurrently dorsoventral and anteroposterior compartments of the primordium are patterned. Despite large morphological differences between body appendages of different phyla, it has been found that homologous or orthologous genes are often involved in similar regulatory processes during body appendage formation. The regulatory gene networks controlling the formation and shaping of body appendages during embryogenesis have so far been studied in detail in dipteran insects and vertebrates only. However, formation and patterning of a vertebrate limb differ in many aspects compared with development of the arthropod leg. Vertebrate limbs are mainly formed by mesodermal tissue [1], whereas arthropod legs are epidermal structures [2]. Two regions, organizing the growth and patterning of the vertebrate limb, have been described before: the apical ectodermal ridge (AER) and the zone of polarizing activity (ZPA) [3]. A positive feedback interaction between the AER and the ZPA is important for the development of the limb and has no complement mechanism in arthropods [1]. Other differences involve the number of leg elements and joints, the position of skeletal and muscular elements as well as their innervation. All of these differences may, however, be regarded to be apomorphic traits. Therefore, despite shared aspects of some attributes of developmental gene expression, the appendages of vertebrates are independently evolved and non-homologous as structures in an evolutionary sense.

**Fig. 1:**
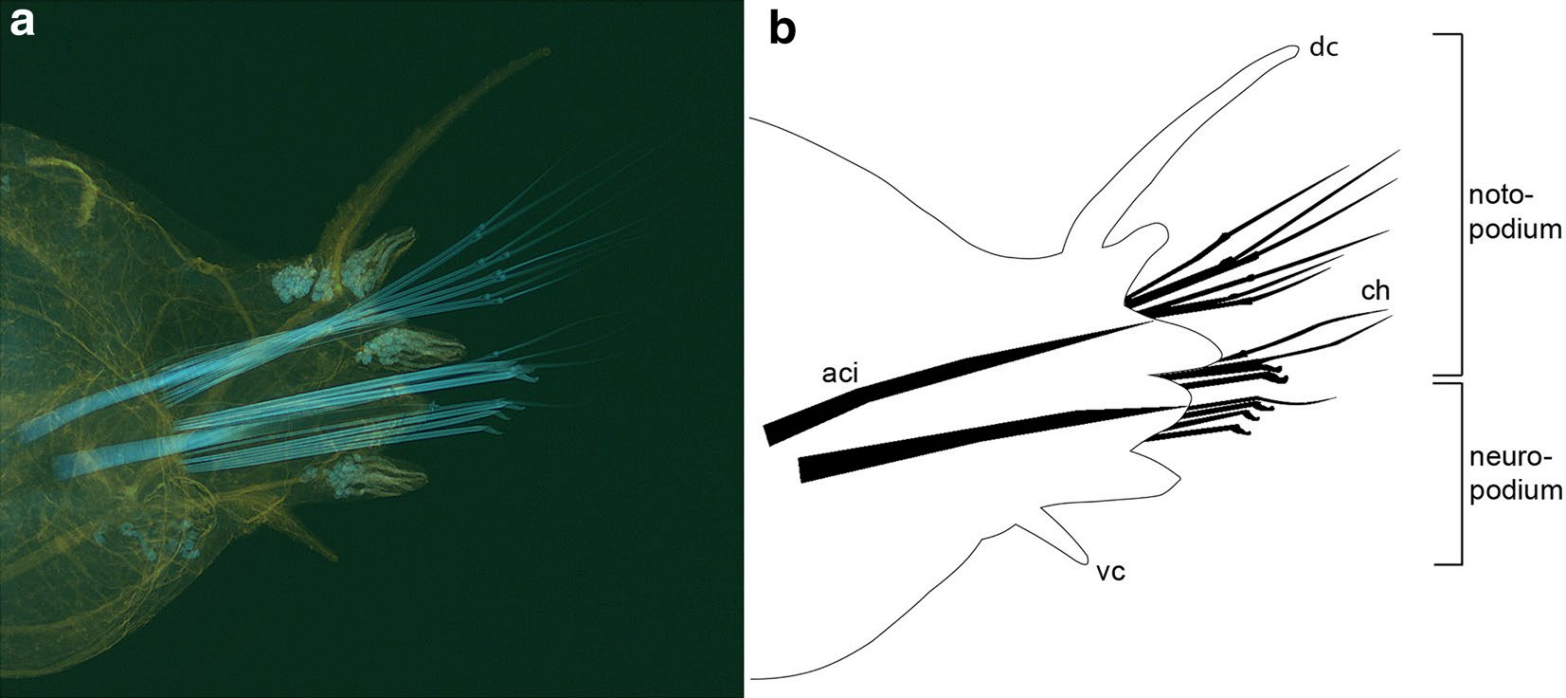
Transverse section through a mature parapodium of *Platynereis dumerilii.* **a** Immunohistochemical labeling of axons with anti-acetylated tubulin antibody (*yellow*). Autofluorescence excited by UV light highlights aciculae and chaetae (*blue*), **b** Schematic of parapodial structures. (*aci*) aciculae, (*ch*) chaetae, (*dc*) dorsal cirrus, (*vc*) ventral cirrus

There is a third, morphologically highly diverse group featuring a variety of different body appendages: polychaete annelids. The most prominent body appendages of polychaetes are bifurcated parapodia flanking the segments of the trunk region. In *Platynereis dumerilii*, these biramous appendages are formed by a dorsal and a ventral branch referred to as noto- and neuropodium. Both bear cirri, the dorsal and ventral cirrus as well as chaetae. The latter project laterally being flanked by spinning glands. Mechanical stability of these lobes is granted by aciculae, stabilizing rods originating in the trunk (Fig. 1). Spatial and temporal expression patterns of several genes known to play important roles in patterning of limb buds in vertebrates and imaginal disks in insects have meanwhile been studied in a variety of representatives of non-dipteran insects and non-insect arthropods [4, 5]. To determine whether these genes are also activated during formation of parapodia and if so to analyze whether these expression patterns show similarities to those observed in other taxa, orthologs of five genes involved in the development of body appendages in arthropods and vertebrates were isolated from the annelid polychaete *Platynereis dumerilii*: homothorax (*Pdu*-*Meis/hth*), extradenticle (*Pdu*-*Pbx1/exd*), Distal-less (*Pdu*-*Dll*), specific protein 1/buttonhead (*Pdu*-*sp/btd*) and decapentaplegic (*Pdu*-*dpp*).

Early expression of *Distal*-*less* (*Dll*) in arthropod appendages starts in broad developmental fields with the distal tip in center, but becomes restricted to the distal region during growth of the appendage. The only exception is the proximally located *Dll* domain present in the insect leg imaginal disk [4, 6]. In contrast to the expression of *Dll*, in non-insect arthropods *homothorax* (*hth*) and *extradenticle* (*exd*) are primarily co-expressed and located in proximal regions of the appendage [4, 7–10]. In *Drosophila*, both proteins seem to be interdependent for stabilization of expression in vivo, but also for nuclear localization of EXD and DNA-binding activity of HTH-EXD [11, 12]. The expression domains of both genes show an interesting aspect. In insects, *exd* is present in all parts of the appendage anlagen, whereas *hth* is limited to the proximal region. However, in non-insect arthropods the size of the expression domains of these two genes is reversed. Prpic et al. demonstrated this reversal of spatial expression in the spider *Cupiennius salei* and described it as an evolutionary shift [8]. Expression of *Dachshund* (*dac*) during arthropod development is located between the expression domains of the proximally and distally expressed genes forming a well-conserved intermediate domain [7–9, 13–15] partially overlapping with the *Dll* domain [6]. For *Drosophila*, Estella et al. showed that *buttonhead* (*btd*) and *specific protein 1* (*sp1*) are required for *Dll* expression in the future leg primordia [16]. In a later publication, this statement was modified. *Btd* seems to play a more insignificant role compared with *sp1* during early leg development. However, both genes are required in later development [17]. Expression of *decapentaplegic* (*dpp*) does not seem to be conserved throughout the arthropods like the previously mentioned genes, and their patterns are by no means similar [4].

The *hth* homolog *Meis* disappears from the distal-most parts as the vertebrate limb grows and regresses to proximal regions during elongation of the limb [18, 19]. Interestingly comparable to the situation in insects, co-expression of the *hth* and *exd* homologs *Meis* and *Pbx* and the formation of heterodimeric complexes of MEIS and PBX [19] was observed in vertebrates. The *Dll* homologs in vertebrates form the *Dlx* gene family. These genes were initially found in the ectoderm of the limb bud. Later, *Dlx* expression is limited to the AER, the region located at a distal rim of the limb anlagen [20]. *Sp8* and *Sp9* are the vertebrate homologs of the arthropod *btd* and *sp1* genes [17, 21]. At the beginning of the development, *Sp8* and *Sp9* are expressed in the ectoderm of the limb. Later on, both are found exclusively in the AER. They regulate *Fgf8* expression, which in return is required for the expression of *Dlx* in the distal limb [21]. *Dach1* is the vertebrate homolog of *dac* and is expressed during the initial phase of limb formation in medial and distal parts of the mesenchyme including the AER, but is not expressed in the posterior distal region. During a later stage of limb development, all parts of the mesenchyme and the AER show *Dach1* expression [22].

Analysis of transient gene expression in forming segments of *Platynereis dumerilii* during normal growth by segment addition from the posterior growth zone region is very difficult. Normal segment formation is neither synchronized within the *Platynereis* culture nor is it predictable. Even animals of exact same age show great variations in size and length and also in growth rates. In addition, segment formation during non-regenerative growth is extremely fast, activating the genes participating in this process only transiently and in an extremely short time frame (minutes to hours compared with days during caudal regeneration). Therefore, formation of posterior structures and activation of genes is studied during caudal regeneration. The favorable conditions of rapid segment growth and body appendage morphogenesis during caudal regeneration allow a thorough and continuous survey of expression patterns.

## Methods

### *Platynereis dumerilii* culture

A laboratory culture of *Platynereis dumerilii* is maintained using methods previously described [23]. In preparation, 3-month-old animals were sedated in 3.75 % MgCl_2_ in natural seawater (NSW). The posterior part was amputated at the 30th segment of the body with a razor blade. Regenerating animals were separated from the culture in dishes in a mixture of natural and artificial seawater (1:1 v/v) and fed with algae and spinach.

Regeneration of posterior ends occurs spontaneously within days after the amputation of posterior trunk regions. The pace at which regeneration occurs varies slightly among specimens of even the exact same age. Two days prior to harvesting the regenerated ends, the worms were starved to empty their digestive tracts. Caudal regenerates were collected 16 days after amputation (days post-amputation, dpa) by cutting at the rostral segment boundary of the last mature (non-regenerated) segment. Regenerated ends were fixed in 3.7 % formaldehyde in phosphate-buffered saline (PBS) overnight at 4 °C, dehydrated in a methanol/PBS series and stored in methanol at −26 °C.

### Cloning and riboprobe synthesis

Fragments of genes were isolated from *P. dumerilii* by degenerated primer PCR on cDNA of mixed larval stages (24, 48 and 72 h post-fertilization). Large 5′ and 3′ cDNA fragments for synthesis of riboprobes were produced by rapid amplification of cDNA ends (RACE) using the SMART RACE cDNA Amplification Kit (BD Bioscience, Heidelberg, Germany) and larval cDNA as template. RACE fragments were cloned into the pGEM-T_easy_ vector (Promega, Mannheim, Germany) and were sequenced by StarSEQ (Mainz, Germany) or Seqlab (Göttingen, Germany). Via PCR with oligonucleotides against SP6 and T7 promotor regions, linear templates for synthesis of riboprobes were generated. Digoxigenin-labeled riboprobes were generated by in vitro transcription using the MEGAscript High Yield Transcription Kit (Ambion, Austin, USA) and digoxigenin-11-UTP (Roche, Mannheim, Germany).

### Whole-mount in situ hybridization

Whole-mount in situ hybridization was performed as previously described by Seaver and Kaneshige for *Capitella teleta* with minor modifications for regenerated ends of *Platynereis dumerilii* [24, 25]. A working concentration of 3 ng/µl was applied for all riboprobes. Results were analyzed with differential interference contrast optics on an Olympus BX 51 microscope and documented with a Nikon Coolpix 4500 camera. The detailed protocol is available upon request.

### Analyses of expression patterns

The rather complex structure of parapodia of nereids including neuro- and notopodia protruding laterally and cirri projecting ventrolaterally and dorsolaterally demands analysis of expression patterns in all three dimensions. To determine the position of expression domains along the anteroposterior axis of the animal as well as along the proximodistal axis of dorsal and ventral cirri, some of the regenerated ends previously subjected to in situ hybridization were mounted ventral or dorsal side up. Ventral or dorsal focal planes enabled the analysis of staining in the cirri including the anal cirri newly formed by the pygidium. Regenerated segments are in different stages of maturation, thus allowing the observation of changes in expression patterns during parapodial growth in a single regenerated end. To assess the location of expression domains in relation to the dorsoventral axis, some regenerated ends were cut into single segments by cutting at the segment boundaries with a scalpel. Segments in different phases of maturation and parapodial growth were subsequently mounted cut face down and analyzed.

### Phylogenetic analysis

Initially, putative orthology of sequences isolated by PCR and RACE was assigned by BLASTX searches of the GenBank database from NCBI. Subsequently, amino acid alignments of highly conserved regions were generated using BioEdit (version 7.2). Prottest (version 3.4) was used to identify the best-fit model of protein evolution for each set of data: JTT+G for *pbx1/exd*, JTT+G+F for *Meis/hth* and JTT+I+G+F for *sp/btd*. Bayesian phylogenetic analyses were conducted using MrBayes 3.2.6. A consensus of 1000 trees was calculated for each gene. Resulting trees were displayed with FigTree 1.4.2 and are presented in Additional file 1.

## Results

### *Pdu*-*Dll* expression during regeneration

**Fig. 2:**
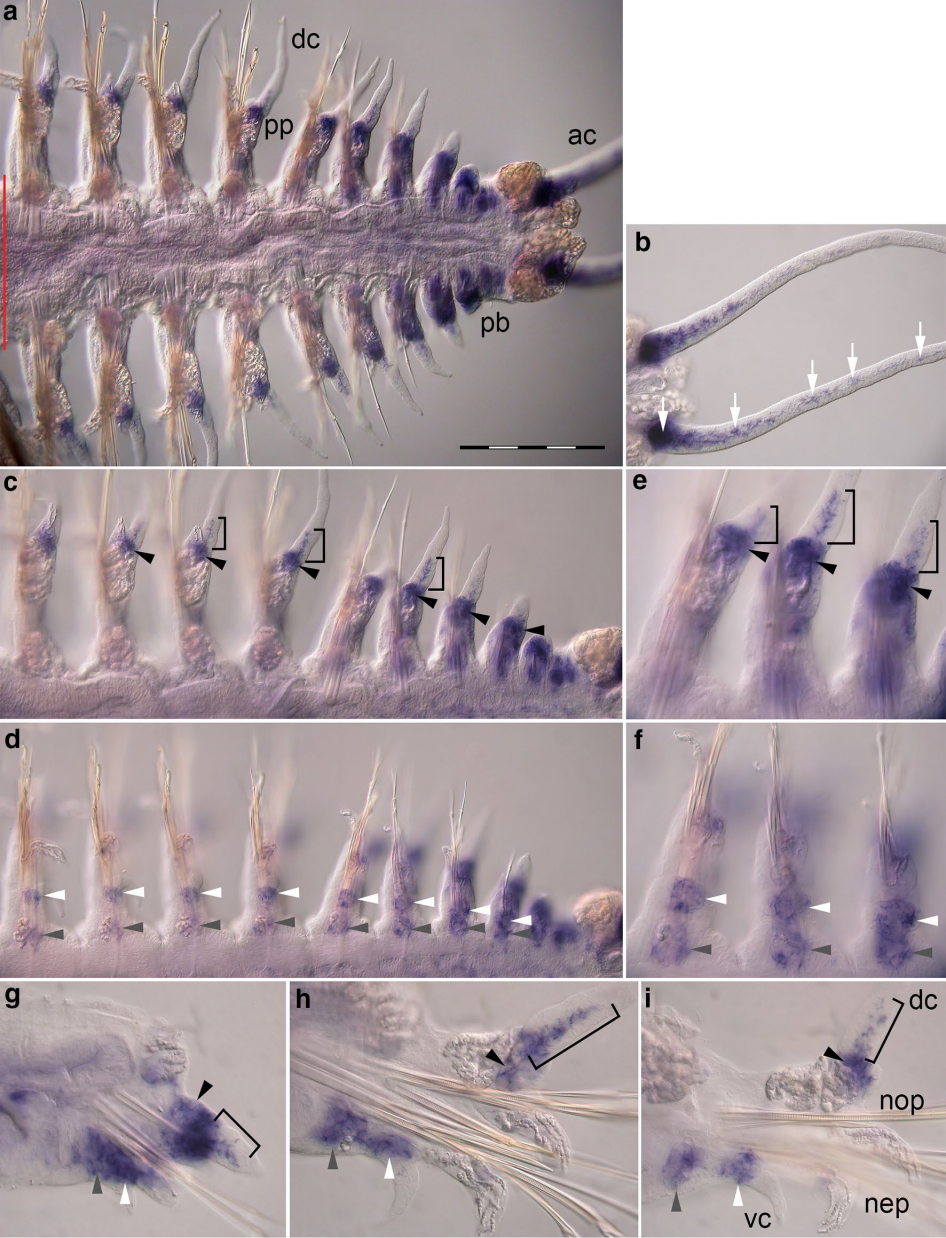
Expression of *Pdu*-*Dll* during caudal regeneration of *Platynereis dumerilii* [16 dpa (days post-amputation)]. In situ hybridizations, anterior is to the *left* in (**a**–**f**). **a** Ventral view of the regenerated posterior end. The *red line marks* the site of amputation. Regenerated tissues are to the *right*. **b** Expression is located at the base of and in the anal cirri (*white arrows*), ventral view (**c**, **e**) *Pdu*-*Dll* expression in the notopodia at the base of the dorsal cirri (*black arrowheads*) and in the proximal part of the dorsal cirri (*black brackets*) (**d**, **f**) Developing neuropodia show expression at the base of the ventral cirri (*white arrowheads*) and at the base of the parapodia (*gray arrowheads*). **g**–**i** Optical transverse sections of segments in different phases of maturation with focus on parapodia, ventral side down. Expression domains indicated in **c**–**f** are labeled accordingly, (*ac*) anal cirri, (*dc*) dorsal cirrus, (*nep*) neuropodium, (*nop*) notopodium, (*pb*) parapodial bud, (*pp*) parapodia, (*vc*) ventral cirrus. *Scale bar* equals 200 µm

Expression of *Pdu*-*Dll* can be detected in several parts of the parapodium, of the regenerating trunk and within the anal cirri of the newly formed pygidium (Fig. 2a). These expression domains are present in all of the regenerated segments but fade during maturation of the new segments leaving the oldest, i.e., anteriormost ones with the weakest signal. The proximal regions of the anal cirri show broad expression of *Pdu*-*Dll* with additional labeled cells located within the anal cirri (Fig. 2b). The notopodium exhibits *Pdu*-*Dll* expression in a domain located at the base of the dorsal cirrus (Fig. 2c). In addition, 8–10 cells located in the center of the dorsal cirrus are labeled (Fig. 2e, h). In the neuropodium, an expression domain is present at the base of the parapodium. During parapodial differentiation and growth, this domain splits into two smaller subdomains. One remains at the base of the parapodium, and the other one is relocated to the base of the ventral cirrus (Fig. 2d, f). However, no expression of *Pdu*-*Dll* is found in the ventral cirrus. In mature segments of *Platynereis dumerilii*, expression of *Pdu*-*Dll* could not be detected (not shown).

### *Pdu*-*dpp* expression during regeneration

**Fig. 3:**
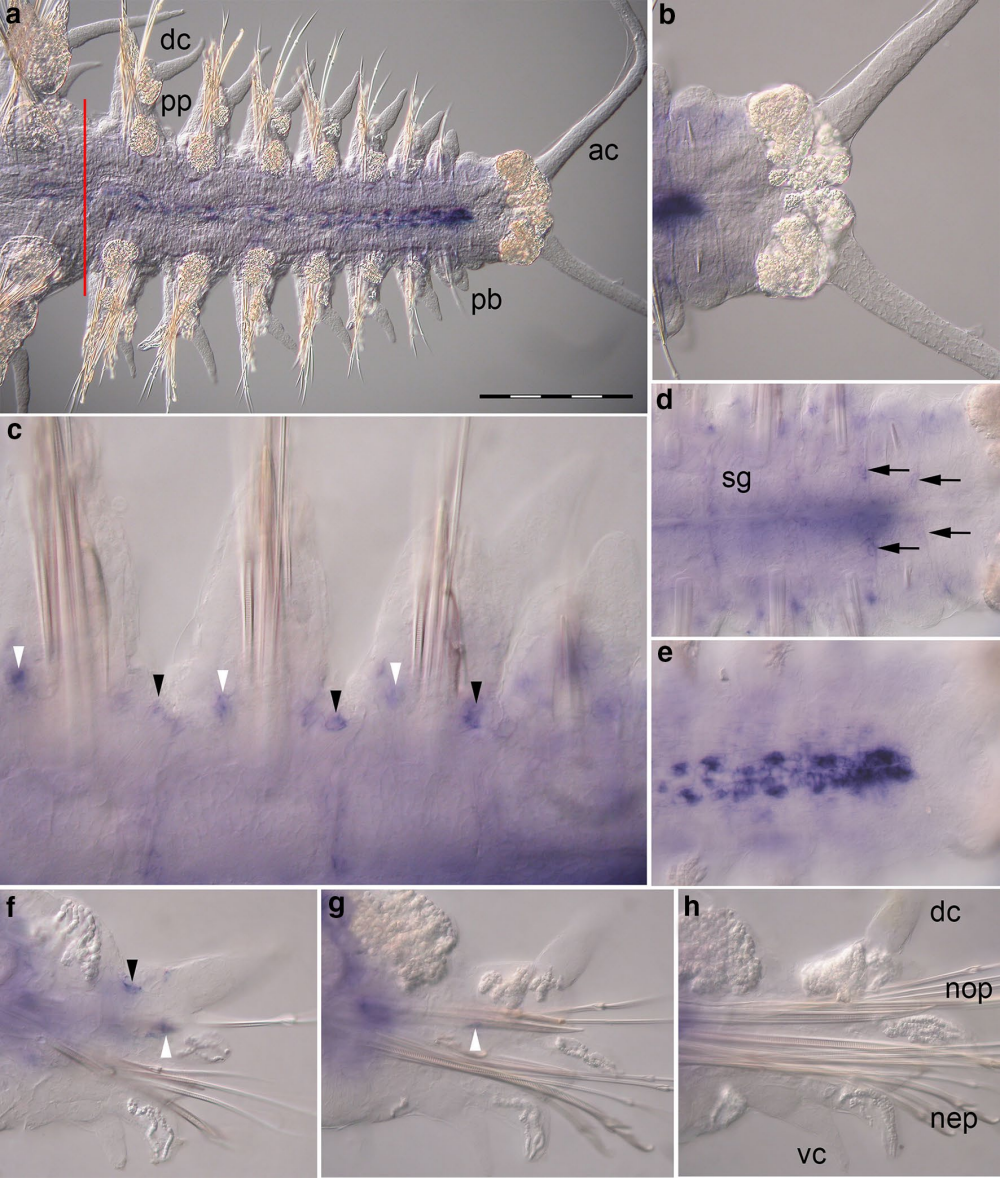
Expression of *Pdu*-*dpp* during caudal regeneration of *Platynereis dumerilii* (16 dpa). In situ hybridizations, anterior is to the *left* in (**a**–**e**). **a** Regenerated posterior end, ventral view. The *red line marks* the site of amputation. Regenerated tissues are to the *right*. The last mature segment is on the *left side*. **b** No expression of *Pdu*-*dpp* in the pygidium, posterior growth zone region and anal cirri (ventral view). **c** *Pdu*-*dpp* is expressed dorsally in the notopodium at the segment boundary (*black arrowheads*) and in the lower notopodium (*white arrowheads*). **d** Ventral expression in the trunk region near the segment boundary (*black arrows*). **e** *Pdu*-*dpp* expression in cell clusters on the dorsal side of the youngest segments. **f**–**h** Optical transverse sections of segments at different stages of maturation with focus on parapodia, ventral side down. Expression domains indicated in (**c**–**e**) are labeled accordingly (*ac*) anal cirri, (*dc*) dorsal cirrus, (*nep*) neuropodium, (*nop*) notopodium, (*pb*) parapodial bud, (*pp*) parapodia, (*sg*) segmental boundary (*vc*) ventral cirrus. *Scale bar* equals 200 µm

Expression of *Pdu*-*dpp* is limited to domains in both the parapodia and the trunk consisting of only a few cells each (Fig. 3a). The anal cirri show no expression of *Pdu*-*dpp* (Fig. 3b). In a parapodium, two small expression domains of *Pdu*-*dpp* each consisting of 3–4 cells were labeled. Both domains are located in the notopodium (Fig. 3c, f). Transcripts of *Pdu*-*dpp* can be detected in the proximal part of the notopodium near the segment boundary subepidermally and also more central in the lower notopodium (Fig. 3c, f, g). The expression pattern is transient as both domains disappear during later stages of segment development (Fig. 3g, h). In addition to expression within body appendages, transcripts of *Pdu*-*dpp* were detected in the regenerating trunk. Small groups of 3–4 cells on the ventral side of the trunk seemingly associated with the segmental nerves express *Pdu*-*dpp* (Fig. 3d). The dorsal side of the youngest segments of the trunk exhibits strong subepidermal expression in single cells lateral to the dorsal midline. These cells expressing *Pdu*-*dpp* are located between the dorsal longitudinal muscles near the position of the forming dorsal blood vessel (Fig. 3e). Mature segments of *Platynereis dumerilii* show weak expression of *Pdu*-*dpp* in a few cells flanking the dorsal midline (Fig. 3a), however, no *Pdu*-*dpp* expression in mature parapodia was detected.

### *Pdu*-*sp/btd* expression during regeneration

**Fig. 4:**
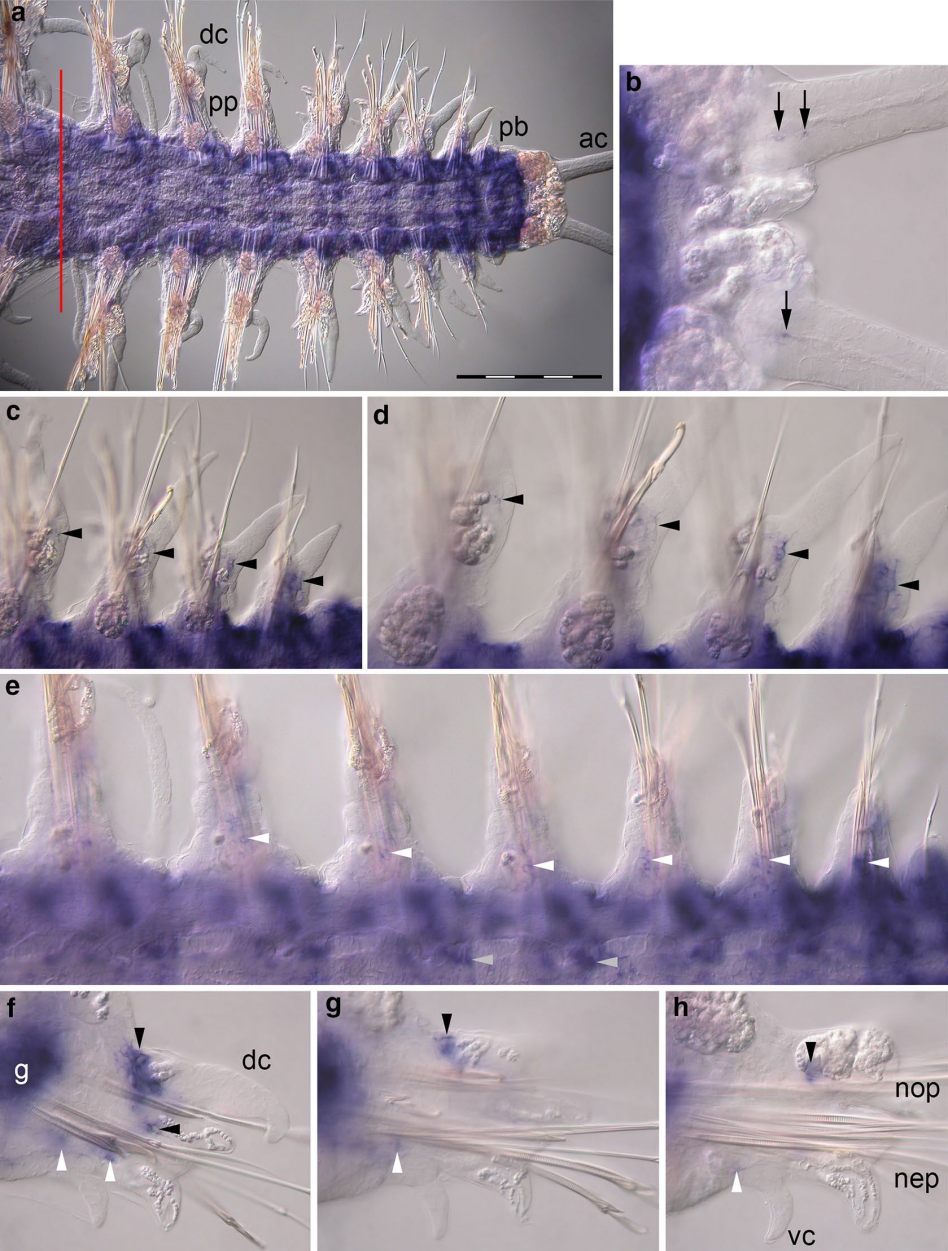
Expression of *Pdu*-*sp/btd* during caudal regeneration of *Platynereis dumerilii* (16 dpa). In situ hybridizations, anterior is to the *left* for (**a**–**e**). **a** Ventral view of the regenerated posterior end. The *red line marks* the site of amputation. Regenerated tissues are to the *right*. The last mature segment is on the *left side*. **b** Ventral view of *Pdu*-*sp/btd* expression in single cells at the base of the anal cirri (*black arrows*). **c**, **d** Shifting expression domains of *Pdu*-*sp/btd* in developing notopodia (*black arrowheads*). **e** *Pdu*-*sp/btd* exhibits expression at the base of developing neuropodia (*white arrowheads*). An additional expression domain is located in the central nervous system near the segment boundary *light* *gray arrow heads*). The gut tissue exhibits moderate *Pdu*-*sp/btd* expression. **f**–**h** Optical transverse sections of segments in different stages of maturation with focus on parapodia, ventral side down. Expression domains indicated in **c**–**e** are labeled accordingly (*ac*) anal cirri, (*dc*) dorsal cirrus, (*g*) gut, (*nep*) neuropodium, (*nop*) notopodium, (*pb*) parapodial bud, (*pp*) parapodia, (*vc*) ventral cirrus. *Scale bar* equals 200 µm

Low-level expression of *Pdu*-*sp/btd* can be detected in the epidermis of the trunk of all regenerated segments including the posterior growth zone region (Fig. 4a). The pygidium is free of *Pdu*-*sp/btd* expression; however, two cells in the proximal region of each anal cirrus expressing *Pdu*-*sp/btd* are labeled (Fig. 4b; black arrows). Within the growing parapodia, expression of *Pdu*-*sp/btd* is found in the proximal part of both noto- and neuropodium. In the notopodium of young segments, a major subepidermal expression domain formed by approximately 12–15 cells per parapodium and a minor expression domain located in the lower notopodium consisting of 4–6 cells per parapodium are visible (Fig. 4c, d). The neuropodium also exhibits small expression domains, located in the center and comparable to the minor expression domain of the notopodium (Fig. 4e, f). In later stages of segment maturation, the overall expression of *Pdu*-*sp/btd* in parapodia is downregulated and dorsal expression domains exhibit a shift in distal direction. This can be observed in the ventral view (Fig. 4d) as well as in transverse sections (Fig. 4g, h). The expression domain in the neuropodium remains in the proximal part of the parapodium (Fig. 4e, g, h). Additionally, we found some paired expression domains within the trunk. One pair is located in the nervous system near the posterior segment boundary. Another expression domain consisting of many cells surrounding the gut is located slightly rostral compared with the expression in the nervous system (Fig. 4e). Mature segments of *Platynereis dumerilii* exhibit low-level expression of *Pdu*-*sp/btd* in the trunk region associated with the gut only (blue staining in the trunk region in Fig. 4a). No expression of *Pdu*-*sp/btd* in mature parapodia can be detected.

### *Pdu*-*Meis/hth* expression during regeneration

**Fig. 5:**
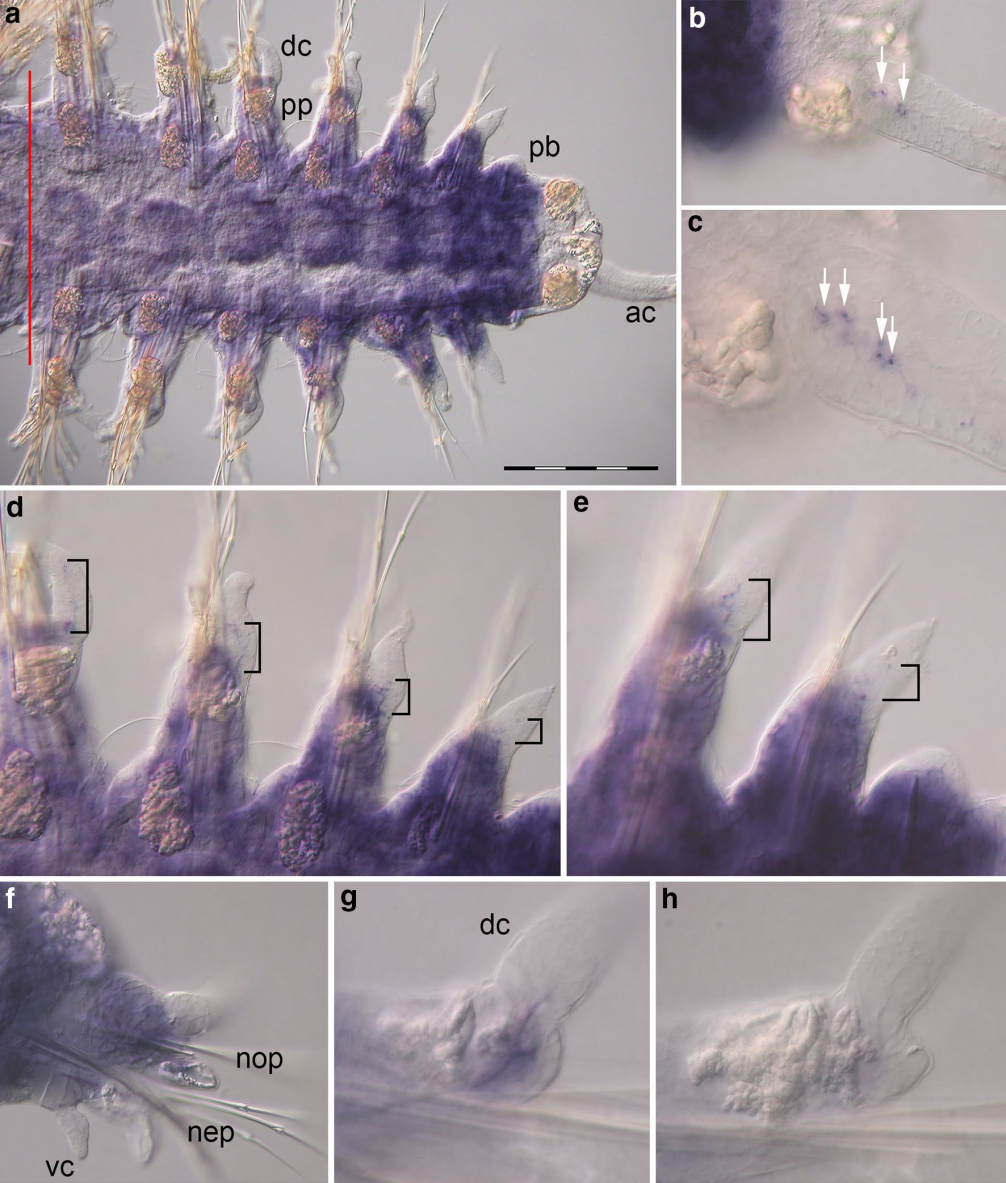
Expression of *Pdu*-*Meis/hth* during caudal regeneration of *Platynereis dumerilii* (16 dpa). In situ hybridizations, anterior is to the *left* for (**a**–**e**). **a** Regenerated posterior end, ventral view. The *red line marks* the site of amputation. Regenerated tissues are to the *right*. The last mature segment is on the *left side*. Strong *Pdu*-*Meis/hth* expression in the coelothel of the forming and maturing segments and subepidermal cells of the posterior growth zone region (*blue staining* in the trunk region). **b**, **c** Expression in the anal cirri is limited to 4–6 cells near the base of the cirri (*white arrows*) Ventral view. **d**, **e** Broad *Pdu*-*Meis/hth* expression in the notopodium. Expression in a few cells in the center of the dorsal cirrus (*black brackets*). **f**–**h** Optical transverse sections of segments in different stages of maturation with focus on parapodia, ventral side down. **g**, **h** show close ups of notopodia. (*ac*) anal cirrus, (*dc*) dorsal cirrus, (*nep*) neuropodium, (*nop*) notopodium, (*pb*) parapodial bud, (*pp*) parapodia, (*vc*) ventral cirrus. *Scale bar* equals 200 µm

During caudal regeneration of *Platynereis dumerilii*, *Pdu*-*Meis/hth* exhibits broad expression in the coelothel of all segments and in the posterior growth zone region (Fig. 5a). The pygidium is free of *Pdu*-*Meis/hth* expression. In the anal cirri, however, small expression domains consisting of approximately 4–6 cells per cirrus located near the base of the cirri are detectable (Fig. 5b, c). *Pdu*-*Meis/hth* is also expressed in large parts of the parapodium (Fig. 5d, e). In early segments, the major expression domain is located in the notopodium, excluding the dorsal cirrus and the distal tips. Though *Pdu*-*Meis/hth* expression is also detectable in the neuropodium, this expression domain is not as extensive as the domain in the notopodium. The main expression in this part of the appendage is limited to the proximal region of the parapodia (Fig. 5f). While broad parapodial expression of *Pdu*-*Meis/hth* decreases during a later phase of parapodial growth, expression in a few cells in the center of the dorsal cirrus is upregulated during this time (Fig. 5d, g). The signal of *Pdu*-*Meis/hth* expression fades toward the end of parapodial growth. In the parapodia of the oldest segments (Fig. 5h), transcripts of *Pdu*-*Meis/hth* cannot be detected. Likewise broad expression of *Pdu*-*Meis/hth* in the coelothel of the trunk is strongest in young segments, decreasing with maturation of the newly formed segments. Low-level expression of *Pdu*-*Meis/hth* in the coelothel of mature segments can be observed; however, *Pdu*-*Meis/hth* expression is absent in mature parapodia (Fig. 5a).

### *Pdu*-*Pbx1/exd* expression during regeneration

**Fig. 6:**
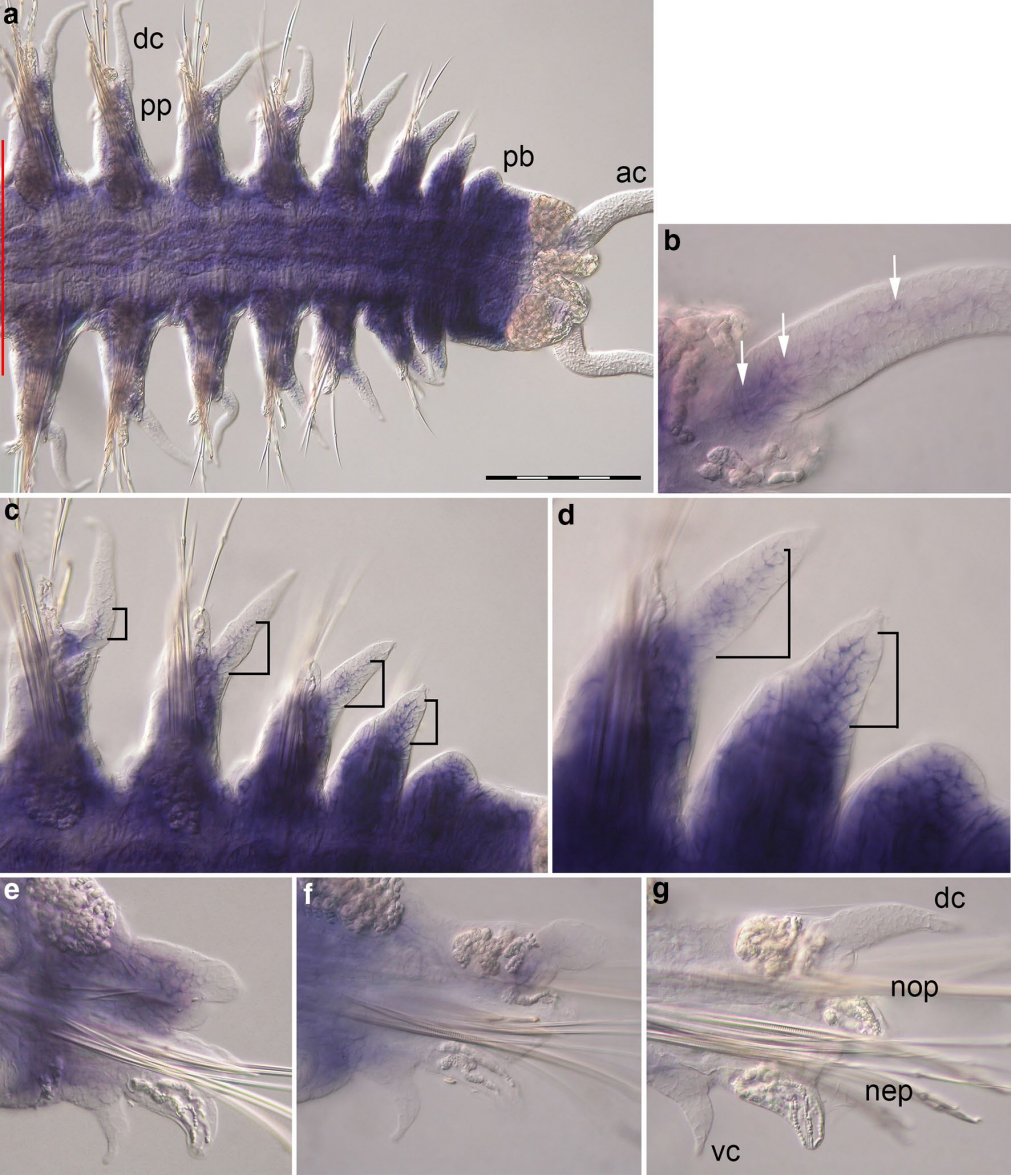
Expression of *Pdu*-*Pbx1/exd* during caudal regeneration of *Platynereis dumerilii* (16 dpa). In situ hybridizations, anterior is to the *left* for (**a**–**d**). **a** Ventral view of the regenerated posterior end. The *red line marks* the site of amputation. Regenerated tissues are to the *right*. *Pdu*-*Pbx1/exd* is expressed in the posterior growth zone region and the coelothel of forming and maturing segments (*blue staining in the trunk*). **b** Cells at the base and in the proximal part of the anal cirri express *Pdu*-*Pbx1/exd* (*white arrows*). **c**, **d** Broad subepidermal *Pdu*-*Pbx1/exd* expression in developing notopodia. *Black brackets* mark expression in the dorsal cirri. **e**–**g** Optical transverse sections of segments in different phases of maturation with focus on parapodia. (*ac*) anal cirri, (*dc*) dorsal cirrus, (*nep*) neuropodium, (*nop*) notopodium, (*pb*) parapodial bud, (*pp*) parapodia, (*vc*) ventral cirrus. *Scale bar* equals 200 µm

**Fig. 7:**
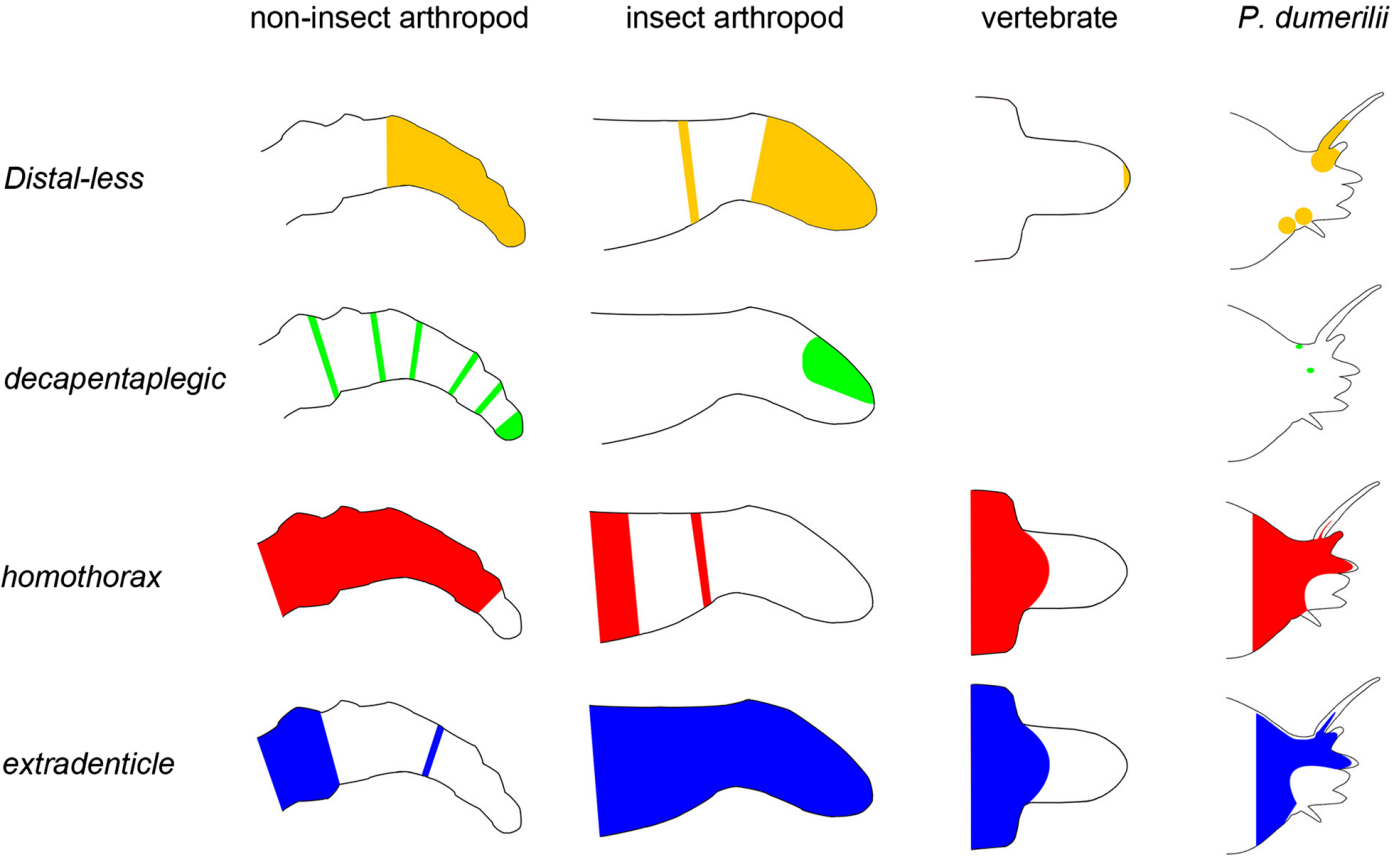
Schematic comparison of spatial gene expression data for *Distal*-*less*, *extradenticle*, *homothorax* and *decapentaplegic* in developing appendages of various taxa. Adapted from [4]. Dynamics of gene expression have been ignored [4, 5, 46]

The extradenticle ortholog of *Platynereis dumerilii* is expressed in the coelothel of all regenerated segments including the posterior growth zone region (Fig. 6a). The anal cirri exhibit expression of *Pdu*-*Pbx1/exd* as well. This expression domain is primarily located at the bases of the anal cirri surrounded by the posterior spinning glands with a few labeled cells detectable in more distal regions of the cirrus (Fig. 6b).The main parapodial expression domain of *Pdu*-*Pbx1/exd* is located in the notopodium in tissues underneath the epidermis (Fig. 6e). A smaller expression domain of *Pdu*-*Pbx1/exd* is detectable in the proximal part of the neuropodium. This gene is expressed in the dorsal cirri from the onset of their development with the expression domain expanding slightly in distal direction with growth of the cirrus (Fig. 6c, d). During later stages of parapodial growth, expression in all regions of the parapodium fades (Fig. 6f) and finally disappears in older segments (Fig. 6g). Expression of *Pdu*-*Pbx1/exd* in mature segments is identical to the previously described expression pattern of *Pdu*-*Meis/hth* in mature segments (thus not shown). Transcripts of *Pdu*-*Pbx1/exd* are detectable as persisting low-level expression in the coelothel of mature segments. *Pdu*-*Pbx1/exd* expression is absent in mature parapodia (Fig. 7).

## Discussion and conclusion

The development of body appendages during normal development and during the regeneration process does not necessarily require activation of the same subset of genes. Normal development of *Platynereis dumerilii* is well described by Fisher et al. [26]. The development of the egg starts with spiral cleavage and leads to a first larval stage—the trochophore larva. This trochophora and the subsequent metatrochophore larva possess the typical prototroch used for locomotion; the trunk already possesses the anlagen of the first setigerous segments—three pairs of so-called setal sacs [27, 28] that can be regarded as the imaginal disks of the parapodia. During larval development, these anlagen shift outward and form protruding parapodial buds and setae. Soon afterward, the typical three-segmented young worm—the nectochaete stage—starts first active crawling movements utilizing these parapodia. These early larval segments thus develop simultaneously and are referred to as deutomeres, whereas all subsequent segments develop iteratively from a posterior growth zone region and are called tritomeres. *Platynereis dumerilii* is capable of caudal regeneration [23, 29], i.e., the loss of a posterior region of the trunk can be regenerated completely. During regeneration, a blastema is formed at the posterior end. While some of the newly formed tissues derive from blastemal cells, others grow into the regenerating end from the posterior stump. The nervous system is built by an outgrowth of the remaining nerve cord in the remaining segments in the stump [25, 30]. Wattez-Combaz demonstrated that the outgrowing nervous system is the driving force for dorsoventral morphogenesis within the regenerating segments [31]. The developmental processes of parapodial morphogenesis in deutomeres, tritomeres as well regenerating segments thus vary, and our results clearly show this for the expression of a typical subset of genes that are also involved in body appendage formation in normogenesis of *Platynereis*, other annelids and arthropods.

*Pdu*-*Dll* expression in young parapodial buds of polychaetes shows similarities to the expression of *Dll* in appendage primordia during early development of arthropods and vertebrates. The subsequent relocation of the expression domain in distal direction as seen in these taxa [32] can also be observed in the polychaete annelid *Platynereis dumerilii*. Two of three main expression domains of *Pdu*-*Dll* are shifting distally during the ongoing morphogenesis of the segment, these domains persisting at the bases of the dorsal and ventral cirrus, respectively. No expression, however, is found in the distal-most part of the cirri. In arthropods and vertebrates, the *Dll* expression domain generally extends into the distal tips of appendages in all developmental stages [4]. The third expression domain of *Pdu*-*Dll* remains in a proximal position. These results correspond to the work of Winchell et al. on the polychaete *Neanthes* [33]. During parapodial growth, *Pdu*-*Dll* seems to be expressed in an atypical way not explicitly indicating a role in distal patterning of the appendage.

*Sp1* and *btd* in arthropods as well as the vertebrate orthologs *sp8* and *sp9* are both required for *Dll* expression in the arthropod and vertebrate leg primordia, respectively. Therefore, expression of these genes can be detected primarily in distal regions of the developing appendage [16, 17, 21]. In *Platynereis dumerilii*, expression of *Pdu*-*sp/btd* seems to be related to *Pdu*-*Dll* expression. At first, in young segments *Pdu*-*sp/btd* expression is found in the proximal part of the parapodia like *Pdu*-*Dll*. During ongoing segment morphogenesis, a shift in distal direction comparable to the shift of *Pdu*-*Dll* expression can be observed. The weaker expression and the final position of *Pdu*-*sp/btd* after the shift differ from *Pdu*-*Dll* expression in the notopodium. This could be explained by the function of sp/btd activating Dll. In older segments, the expression of *Pdu*-*Dll* is terminated and likewise *Pdu*-*sp/btd* is downregulated. In the neuropodium, no shift in distal direction was observed. The neuropodium shows a persisting proximal expression domain of *Pdu*-*Dll* and expression of *Pdu*-*sp/btd* at the same position. In addition, expression of *Pdu*-*sp/btd* is not downregulated. We thus show that spatial expression of both genes exhibits some striking similarities during development of the appendage. The interaction between *Pdu*-*sp/btd* and *Pdu*-*Dll* and a resulting role in patterning of the parapodia, however, can only be assumed. Additional experiments (e.g., knockout) could clarify whether *Pdu*-*sp/btd* has the same function as in arthropods and vertebrates or is involved in a completely different developmental process.

In *Drosophiladpp* is involved in the process of determining the anterior–posterior boundary [34, 35], the dorsal and ventral regions of the imaginal disks [36] and the proximodistal axis [6, 35, 37] of the appendage. In other insect arthropods, expression of *dpp* orthologs is also found in the limb buds. However, this expression is not comparable to the situation in *Drosophila* and may not have the same function during appendage development [4, 38, 39]. Angelini and Kaufman suggested that a function of *dpp* in anteroposterior axis formation is conserved, while determination of dorsoventral and proximodistal axis by *dpp* in other insect arthropods is unlikely [4]. Expression of the polychaete ortholog *Pdu*-*dpp* is detectable exclusively in the notopodium, the dorsal part of the parapodium. This is similar to *dpp* expression in the leg imaginal disk of *Drosophila* where hedgehog signaling leads to dorsal *dpp* expression on the anterior side of the anterior–posterior boundary of the disk [34]. This might hint toward a role of *Pdu*-*dpp* in defining the anterior–posterior axis of appendages in *Platynereis dumerilii*. However, expression domains of *Pdu*-*dpp* are rather small making it hard to assume they might set clear boundaries in developing parapodia. To confirm or falsify this hypothesis, parapodial expression of other possible anterior–posterior-related genes like *wingless* and *hedgehog* orthologs must be investigated in *Platynereis* in detail.

*Hth* and *exd* and their vertebrate orthologs *Meis* and *Pbx* are typically expressed in proximal regions of the developing appendages [4, 19]. Spatial expression of these genes is reversed in higher insects compared with the expression domains observed in non-insect arthropods [4, 7–10, 38]. In *Platynereis dumerilii*, expression of *hth* and *exd* is not restricted to the proximal region. Both genes are activated in nearly all regions of the parapodia, excluding the distal part of the neuropodium and the distal tip of the notopodium. Considering the expression in non-insect and insect arthropods, one hypothesis might be an ancestral form of spatial *Meis/hth* and *Pbx1/exd* expression with both genes being active all over the appendage primordium. Another possibility for these broad expression domains, however, could be a different function in the developmental process of parapodia compared with that in arthropods or vertebrates. Further functional experiments are needed to reveal the function of *hth* and *exd* in formation of annelid appendages.

The question regarding the evolutionary relationship between polychaete parapodia and arthropod limbs has been the topic in several previous publications and lead to an extensive discussion (e.g., [40]). Homology is classically defined as an “historical continuity in which morphological features in related species are similar in pattern or form because they evolved from a corresponding structure in a common ancestor” [41]. Recently, the new definition of “deep homology” has been coined for such cases in which continuity may not directly be obvious (i.e., regulatory mechanisms during development of appendages) [41]. First of all, parapodia and arthropod limbs show no structural similarities except for the basic fact that both develop from ventrolateral buds [42]. Prpic proposed that the early body organization including the limb primordia of the brine shrimp *Artemia franciscana* is identical to the early development in *Platynereis* [43–45]. He deduced that both structures are homologous to each other [43]. The primary goal of this study, however, was to determine whether orthologs of genes known to play key roles in patterning of body appendages during development in arthropods and vertebrates are expressed in parapodial primordia during formation of these appendages during caudal regeneration of a polychaete. The second question was whether the patterns observed could be similar to what was shown in arthropods. The morphology of parapodia, however, is rather complex, not only featuring a single proximodistal axis and involving additional regions, as, e.g., dorsal and ventral cirri and several lobes. Expression patterns can thus not easily be compared across phyla. The previously coined “deep homology” hypothesis, however, can not easily be substantiated by results derived from spatial expression data alone. To achieve more clarity, ultimately functional analyses of these genes along with comparisons of the resulting networks are needed to understand their roles during parapodial formation.

The genes analyzed in this study exhibit expression in forming segments including parts of parapodial buds during caudal regeneration of the polychaete *Platynereis dumerilii* indicating possible roles in formation of the body appendages of annelids. In comparison with data from mostly arthropod clades, however, both similarities and differences of gene expression can be observed. Questions concerning the homology of body appendages across the now obsolete group Articulata, however, could not be answered. The positions at which primordia of segmentally iterated body appendages are formed in arthropods and annelids are not identical. In *Platynereis*, a segmental compartment exhibits *engrailed* (*en*) expression at the anterior segmental boundary and expression of *wingless* (*wg*) at the posterior segmental boundary. The parapodial anlagen occupy a mid-segmental position including *wnt1/wingless* expression at the posterior side of the parapodial base [44]. In insects, transient parasegmental boundaries are established during embryogenesis showing the same distribution of *en*/*wg* expression as seen in nereids. However, imaginal disks of insects form at the position of these parasegmental boundaries. Subsequent resegmentation then shifts these anlagen slightly anteriorly.

Representing a toolkit for the patterning of additional body axes, genes have been recruited during evolution to participate in and control the formation of body appendages in general. The high flexibility of this toolkit leads to varying spatial and temporal expression patterns in different body plans and thus differing results across various taxa not necessarily reflecting phylogenetic relationships.

## Additional file

10.1186/s13227-016-0046-6 Phylogenetic analysis of the three new sequences isolated from *Platynereis dumerilii* and related sequences from GenBank using the Bayesian inference model.
