# Supplementary material for: Formation of body appendages during caudal regeneration in Platynereis dumerilii: adaptation of conserved molecular toolsets
Source: EvoDevo. 2016 Apr 12;7:10. doi: 10.1186/s13227-016-0046-6 (PMC4830062; doi:10.1186/s13227-016-0046-6)
Supplement: Supplementary file 1 — 10.1186/s13227-016-0046-6 Phylogenetic analysis of the three new sequences isolated from Platynereis dumerilii and related sequences from GenBank using the Bayesian inference model. [file 13227_2016_46_MOESM1_ESM.pdf]

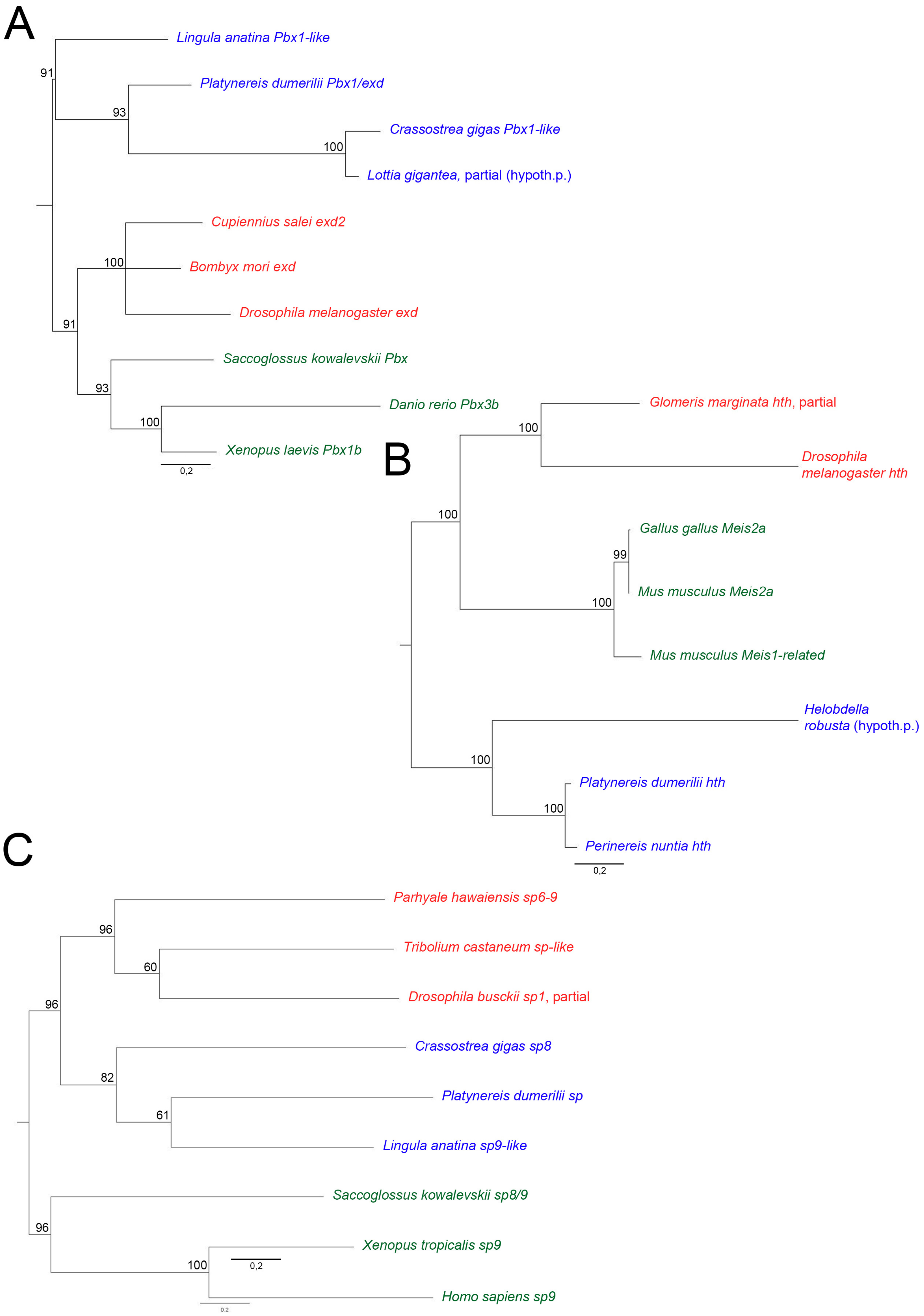
A
Lingula anatina Pbx1-like
Platynereis dumerilii Pbx1/exd
Crassostrea gigas Pbx1-like
Lottia gigantea, partial (hypoth.p.)
Cupiennius salei exd2
Bombyx mori exd
Drosophila melanogaster exd
Saccoglossus kowalevskii Pbx
Danio rerio Pbx3b
Xenopus laevis Pbx1b
91
93
100
100
91
93
100
0,2
B
Glomeris marginata hth, partial
Drosophila melanogaster hth
Gallus gallus Meis2a
Mus musculus Meis2a
Mus musculus Meis1-related
Helobdella robusta (hypoth.p.)
Platynereis dumerilii hth
Perinereis nuntia hth
100
100
99
100
100
100
0,2
C
Parhyale hawaiensis sp6-9
Tribolium castaneum sp-like
Drosophila busckii sp1, partial
Crassostrea gigas sp8
Platynereis dumerilii sp
Lingula anatina sp9-like
Saccoglossus kowalevskii sp8/9
Xenopus tropicalis sp9
Homo sapiens sp9
96
60
96
82
61
96
100
0,2

Additional file 1: Phylogenetic analysis of the three new sequences from *Platynereis dumerilii* and related sequences from GenBank using the Bayesian inference model. Prottest was used to identify the best-fit model of protein evolution for each set of data (JTT+G for exd, JTT+G+F for hth and JTT+I+G+F for sp). Deuterostome sequences in green, lophotrochozoan sequences in blue and ecdysozoan sequences in red. (A) Unrooted tree for *exd* sequences. Complete scientific names and corresponding accession numbers, in order from top to bottom, are: *Lingula anatina* / [NCBI:XP_013405202], *Platynereis dumerilii* / [GenBank:KU249166], *Crassostrea gigas* / [NCBI:XP_011452234], *Lottia gigantea* / [NCBI:XP_009044535], *Cupiennius salei* / [GenBank:CAD57738], *Bombyx mori* / [NCBI:NP_001296565], *Drosophila melanogaster* / [NCBI:NP_523360], *Saccoglossus kowalevskii* / [NCBI:NP_001158431], *Danio rerio* / [GenBank:AAI62483], *Xenopus laevis* / [GenBank:AAI06423], (B) Unrooted tree for *hth* sequences. Complete scientific names and corresponding GenBank accession numbers, in order from top to bottom, are: *Glomeris marginata* / [Genbank:CAD82908], *Drosophila melanogaster* / [NCBI:NP_476576], *Gallus gallus* / [GenBank:AED02523], *Mus musculus* / [GenBank:CAA04138], *Mus musculus* / [GenBank:AAB19194], *Helobdella robusta* / [NCBI:XP_009032038], *Platynereis dumerilii* / [GenBank:KU249167], *Perinereis nuntia* / [GenBank:BAM74492], (C) Unrooted tree for *sp* sequences. Complete scientific names and corresponding GenBank accession numbers, in order from top to bottom, are: *Parhyale hawaiensis* / [GenBank:CBH30981], *Tribolium castaneum* / [NCBI:NP_001034509], *Drosophila busckii* / [GenBank:ALC48334], *Crassostrea gigas* / [GenBank:EKC30539], *Platynereis dumerilii* / [GenBank:KU249168], *Lingula anatina* / [NCBI:XP_013395523], *Saccoglossus kowalevskii* / [NCBI:NP_001161661], *Xenopus tropicalis* / [NCBI:NP_001072269], *Homo sapiens* / [NCBI:NP_001138722].
